# Pulmonary embolism in COVID-19: Ventilation and perfusion computed tomography

**DOI:** 10.1016/j.idcr.2020.e00805

**Published:** 2020-05-11

**Authors:** Anna Maria Ierardi, Salvatore Alessio Angileri, Antonio Arrichiello, Letizia Di Meglio, Martina Gurgitano, Giovanni Maria Rodà, Gianpaolo Carrafiello

**Affiliations:** a Radiology Department, Fondazione IRCCS Cà Granda, Ospedale Maggiore Policlinico, Milan, Italy; b Post-graduate School of Radiology, Università degli Studi di Milano, Milan, Italy; c Radiology Unit, European Institute of Oncology, Milan, Italy; d Radiology Department, Fondazione IRCCS Cà Granda, Ospedale Maggiore Policlinico, Milan, Italy; e Department of Health Sciences, Università degli Studi di Milano, Milan, Italy

**Keywords:** COVID 19, Pulmunary embolism, CT angio, CT perfusion, CT ventilation

## Abstract

This is an illustrated case about CT ventilation and perfusion in Covid patient.

A 68−year-old man from Milan, Italy, was admitted at Policlinico Ca’ Granda Hospital, Milan, Italy, with a nine days history of fever and cough and recent onset of dyspnea. Respiratory rate was 20/min and oxygen saturation 89%. Laboratory studies showed lymphocytopenia 048 × 10^9/L, C-reactive protein 9 mg/dL and an arterial-blood gas (ABG) pH 7.42, PCo2 34, PO2 65, Lactates 1, PaO2/FiO2 305 and HCO3- 23. A bedside chest x-ray was performed, showing interstitial pneumonia. The patient was diagnosed with Coronavirus Disease 2019 (COVlD−19) on the basis of RT-PCR analysis of rhinopharyngeal samples in line with the WHO lnterim Guidance from 13 March 2020. He was started treatment with levofloxacin, hydroxychloroquine, corticosteroid and his ventilation was supported with CPAP. After fifteen days the patient developed severe dyspnea and elevation of D-dimer (3233 ng/mL). In the suspicion of pulmonary embolism, the patient was referred for Pulmonary Angiography Computed Tomography (CTPA). CTPA revealed bilateral acute pulmonary embolism. A dedicate software, IntelliSpace Portal 10 (Philips Healthcare, the Netherlands), permitted to evaluate the volume of perfused and ventilated lung (Fig. 1). This system permits to obtain a map of the perfused and ventilated lung areas at the same time as the CT scan is run. The preliminary results obtained with Intellispace allowed the Radiologists to add information to the CT angiogram without the necessity of further imaging examinations (Nuclear Medicine), that can not be performed in these critically-ill patients and in accordance with the biocontainment measures. These informations might help the clinical management of the patients.

**Fig. 1:**
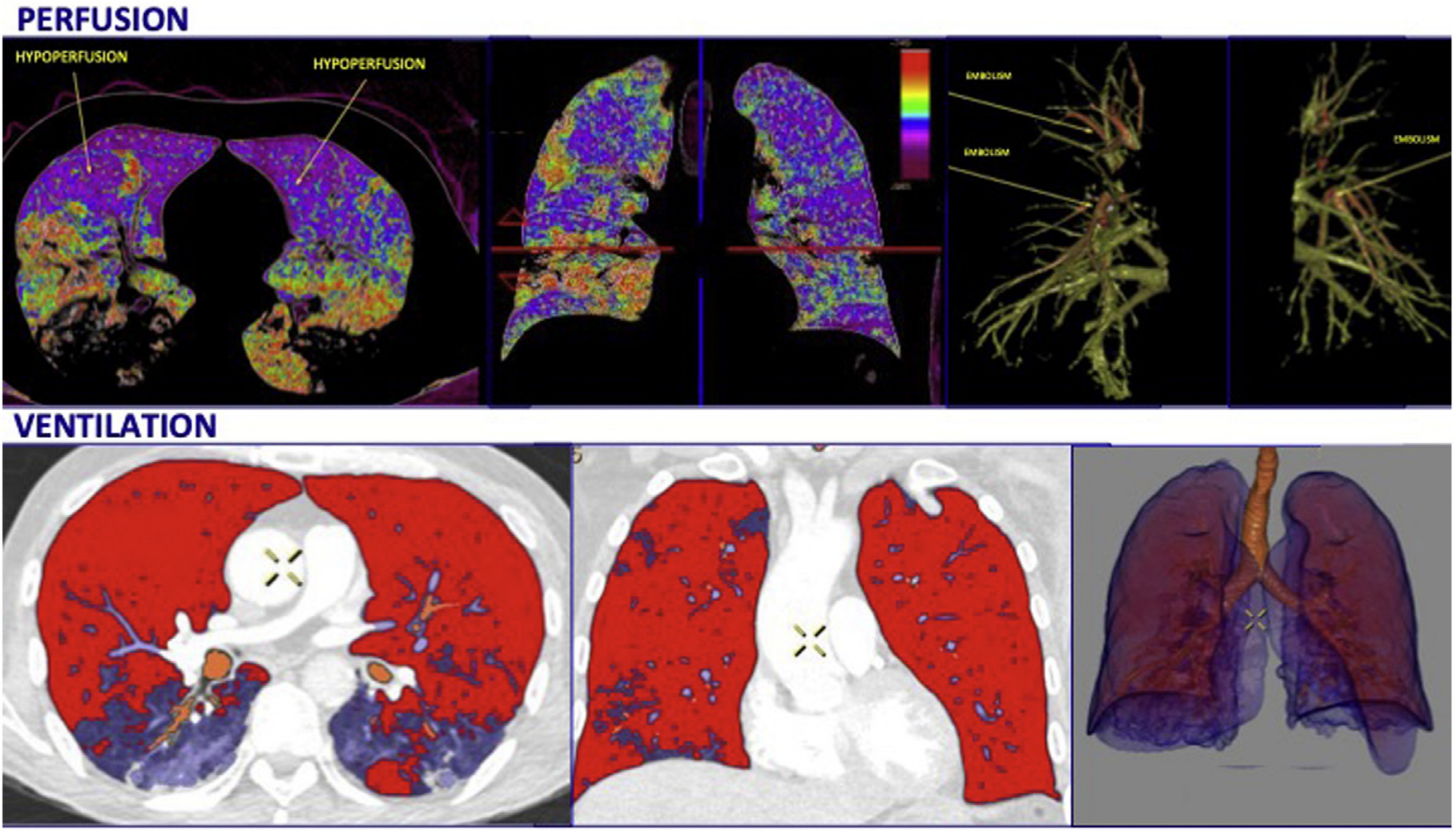
Color maps show the distribution of perfusion and ventilation; perfusion: spectrum from violet to red indicate increasing perfusion: hypoperfusion is indicated by violet areas (corresponding to the area involved by pulmonary embolism). Ventilation: spectrum from red to blue indicate reducing ventilation: blue area indicate impaired ventilation.

Color maps show the distribution of perfusion and ventilation. Red and blue areas indicate normal and impaired ventilation, respectively. Spectrum changing from violet to red indicates increasing perfusion, so the bilateral and multiples violet areas indicate hypoperfusion. In these critical COVID-19 patients with severe ventilation-perfusion mismatch these reconstruction can give more information about the gravity of the ventilation and perfusion alterations. In addition, it allows to locate the exact distribution of the perfusion and ventilation defects and the related mismatch.

## Conflicts of interest

All authors have nothing to disclose

## Sources of funding

No source of funding.

## Consent

Informed consent was obtained from all individual partecipants included in the study.

## Author contribution

All the authors have contributed to conception and design, analysis and interpretation of the data, drafting the article, critical revision and final approval.

## CRediT authorship contribution statement

**Anna Maria Ierardi:** Supervision. **Salvatore Alessio Angileri:** Writing - review & editing. **Antonio Arrichiello:** Writing - review & editing. **Letizia Di Meglio:** Software. **Martina Gurgitano:** Writing - review & editing. **Giovanni Maria Rodà:** Software. **Gianpaolo Carrafiello:** Supervision, Methodology.

